# A Reliability-Based Particle Filter for Humanoid Robot Self-Localization in RoboCup Standard Platform League

**DOI:** 10.3390/s131114954

**Published:** 2013-11-04

**Authors:** Eduardo Munera Sánchez, Manuel Muñoz Alcobendas, Juan Fco. Blanes Noguera, Ginés Benet Gilabert, José E. Simó Ten

**Affiliations:** Instituto de Automática e Informática Industrial (*ai2*), Universitat Politecnica de Valencia, P.O.Box 22012, Valencia, Spain; E-Mails: emunera@ai2.upv.es (E.M.S.); mmunoz@ai2.upv.es (M.M.A.); pblanes@ai2.upv.es (J.F.B.N.); jsimo@disca.upv.es (J.S.T.)

**Keywords:** humanoid robots, self-localization, perception system, particle filter, RoboCup SPL

## Abstract

This paper deals with the problem of humanoid robot localization and proposes a new method for position estimation that has been developed for the RoboCup Standard Platform League environment. Firstly, a complete vision system has been implemented in the Nao robot platform that enables the detection of relevant field markers. The detection of field markers provides some estimation of distances for the current robot position. To reduce errors in these distance measurements, extrinsic and intrinsic camera calibration procedures have been developed and described. To validate the localization algorithm, experiments covering many of the typical situations that arise during RoboCup games have been developed: ranging from degradation in position estimation to total loss of position (due to falls, ‘kidnapped robot’, or penalization). The self-localization method developed is based on the classical particle filter algorithm. The main contribution of this work is a new particle selection strategy. Our approach reduces the CPU computing time required for each iteration and so eases the limited resource availability problem that is common in robot platforms such as Nao. The experimental results show the quality of the new algorithm in terms of localization and CPU time consumption.

## 1. Introduction

The RoboCup SPL is a robotic competition that features soccer matches between two teams of five Nao humanoid robots. The Nao is a small humanoid robot manufactured by the French company Aldebaran Robotics (Paris, France). In this league, the localization system has become as important as any other basic task. Precise information about robots' positions is essential for achieving fluid movements in the field and playing as a team to score goals and win matches. Recent changes in the rules have set the same color for both goals. Until now the two halves of the field could easily be differentiated by checking the color, but this option is no longer available and this task must be handled by the localization system. Thus making self-localization more important in this competition—as has occurred in other areas of robotics where a high degree of autonomy is needed. To obtain a reliable localization system, the kinematic system and sensorial information (inertial, visual, *etc.*) must be adjusted.

In this paper, the implementation of a full localization system is described: ranging from data acquisition to localization itself. All the introduced developments are part of the improvements carried out by the Hidalgos Team that is actively involved in the SPL. Three main goals are achieved:

- Firstly, the implementation of a vision-based measuring system to obtain information regarding robot surroundings. This goal requires a previous study of the camera settings and development of a new software tool for obtaining these settings and evaluating how to compensate for errors.
- The second goal is the definition of a local model system for modeling the sensed surrounding. Auxiliary tools must be implemented that enable the robot to deal with the information provided by the vision system.
- Finally, the main goal is the localization system and global modeling, which must manage the information from the local model to estimate the real position of the robot and its equivalent global model.

This article is organized as follows: in Section 2 the problem of localization and some of the most common solutions are discussed. The following section discusses the architecture used by the Hidalgos Team, while Section 4 describes the main characteristics of its perception system. The developments of the previously referred goals are contained in Section 5 (distance estimation), Section 6 (local modeling) and Section 7 (localization system). In each section, the work performed and the results obtained are carefully described. Conclusions are presented in Section 8.

## 2. The Localization Problem

An increasing number of studies have focused on the localization problem and this has promoted a constant evolution of these systems. Therefore, new localization methods have been developed as existing techniques have been improved.

Early location systems were purely based on odometric readings, but this approach can provide erratic values due to the effect of foot or wheel slippage, or unpredicted slack in the joints in the case of humanoid robots. Moreover, these errors cannot be corrected because of the absence of any feedback, which could help the robot detect its errors. Thus, most sophisticated localization systems make use of sensors to provide such feedback. Sensorial information combined with an appropriate statistical procedure enables an estimation to be made of the robot position with a certain degree of accuracy. The robot soccer teams that participate in the SPL competition have adapted most of their localization systems. In [1] a compilation of the methods used by some of the participant teams can be found—as well their advantages.

One popular method for localization in mobile robotics is the particle filter (PF) and its derivatives. Most implementations are based on the Monte Carlo particle filter (MCL), as described in [2,3]. The MCL method represents an approximation, based on a finite number of random samples (characterized as particles) in the workspace. Each of these particles has an assigned weight corresponding to its probability of matching the observation. Consequently, the belief of each state is determined by a set of tuples:

(1) $$\text{Bel}(x)\approx {\left\{x_{i},w_{i}\right\}}_{i=1,\ldots ,n}$$

This belief distribution is expressed as the output of a Bayes filter that estimates the robot position:

(2) $$\text{Bel}(x_{t})=\frac{p(o_{t}|x_{t},a_{t-1},\ldots ,o)p(x_{t}|a_{t-1},\ldots ,o)}{p(o_{t}|a_{t-1},\ldots ,o)}$$

Normalizing with *n* as a constant:

(3) $$n=p{\left(o_{t}|a_{t-1},\ldots ,o\right)}^{-1}$$

(4) $$\text{Bel}(x_{t})=n.p(o_{t}|x_{t})\int p(x_{t}|x_{t-1},a_{t-1})\text{Bel}(x_{t-1})dx_{t-1}$$

The evolution in time of this set of particles is conditioned by the actions performed by the robot in the specified period of time. The progression of these values in the PF is usually determined by a recursive update through three steps:

(1) Particle distribution update and resampling: in this step each particle *x_i_(t-1)* on the set is updated according to the previous belief distribution and the weights on that iteration:
  (5) $$x_{i}(t-1)∼\text{Bel}(x(t-1))$$
(2) State update: the current set of positions *x_i_(t)* is computed by taking into account the performed action *a(t-1)*, which usually correspond to a displacement of the robot and the previous distribution *x(t-1)*:
  (6) $$x_{i}(t)∼p(x(t)|x(t-1),a(t-1))$$
  According to the sampling/importance resampling (SIR) method, described in [4], the proposed distribution for the current iteration can be expressed as:
  (7) $$q_{t}:=p(x|x_{t-1},a_{t-1})\text{Bel}(x_{t-1})$$
(3) Particle weighting: the proposed distribution *q_t_* expressed in Equation (7) is related with the distribution obtained in the Bayesian filtering procedure expressed in Equation (4), which takes into account the sensorial information (including the observations) in the Equation. As a result of this comparison, the weighting value of each particle involved in the filter can be obtained as follows:
  (8) $$w_{i}=p(o(t)|x_{i}(t))$$
  These weights must be scaled, as the sum never exceeds 1. Thus, the value of the importance characteristics of the ISR method is obtained in each new iteration.

It has been demonstrated in [3] that successive iterations of this algorithm make the original set of particles converge on the distribution *Bel(x)*, in which the number of particles is inversely proportional to the speed of convergence.

This method can be adapted to work with information provided by several types of sensors. In [2,3] the experimental results are obtained using a robot equipped with a laser range sensor combined with a sonar device. Other studies apply this method by using other arrangements of sensors, such as that presented in [5].

However, for our purposes, the application of the MCL using on-board cameras is a preferable option. These on-board cameras can be used as the main perceptive sensors in addition to odometry. The most commonly used types of cameras are omnidirectional or pan and tilt cameras (the cameras in the Nao's head can be rotated via the neck). Several examples are presented in [6], and in [7] up to seven methods are introduced in which the weight of the particles is obtained from visual information.

Focusing on the RoboCup SPL, several participant teams have chosen to use an MCL-based localization system. Most of these implementations attempt to modify the original MCL in order to adapt its operation to this particular environment. One similarity among all these modifications is the definition of how the ‘sensor reset’ procedure is applied. When a ‘lost’ situation is detected by the system (usually produced by a kidnapped robot event or a fall of the robot itself) this procedure enables the filter to be recovered.

The main issue to discuss about the sensor resetting is how to detect a ‘lost’ situation. Improper use of this method will produce an anomalous performance of the filter, leading to new lost situations or wrong estimations. New features were introduced in the filter to achieve sensor resetting only in those cases where no other solution could be applied. The German team B-Human encourages the use of PF localization and validates its application through their achievements in the RoboCup competitions. B-Human proposes an implementation known as the augmented-MCL filter, as described in [8]. In this approach, the resampling method computes the number of particles to be resampled by the inclusion of new parameters, which relate the weighting values with the main goal of increasing or decreasing the effect of the resampling step, as described in the following Equations:

(9) $$W_{\text{fast}}(t)=W_{\text{fast}}(t-1)+\alpha_{\text{fast}}(W_{\text{avg}}-W_{\text{fast}})$$

(10) $$W_{\text{fast}}(t)=W_{\text{fast}}(t-1)+\alpha_{\text{fast}}(W_{\text{avg}}-W_{\text{fast}})$$

(11) $$W_{\text{reset}}=\max(0,1-\frac{W_{\text{fast}}}{W_{\text{slow}}})$$

where *W_avg_* is the average weight of the current iteration, and where *α_fast_* and *α_slow_* are constants that determine the dynamics of the filter. In the iteration, the resampled particles are obtained by a simple comparison between their probabilities and the resetting value obtained in Equation (11) and this approach enables the filter to perform as dynamically as needed.

Other authors have started with the augmented-MCL or adapative-MCL filter and tried to improve the sensory information by adding a multi-observation system, as proposed in [9], to deal with ambiguous landmarks that can induce the assignation of significant weight to wrong particles and thus cause erroneous estimations. In the cited approach, when a sensor reset event turns on, the new particles generated for replacing the old particles will not, as is usually the case, be totally random. Instead, the multi-observation method is taken into account when spreading a new distribution of particles on those locations consistent with the current multi-observation. This enables a quicker convergence to the real position—as long as the information is correct.

At the same level of relevance is the unscented Kalman filter (UKF) based localization system [10]. In the same way as other types of Kalman filters (KF) this technique offers low computational cost and is represented as a normal distribution and parameterized as a Gaussian function. UKF has demonstrated better prediction approximation than other KF modifications by using a deterministic sampling technique to select a minimal set of samples from the observations, as shown in [10]. Typically, the belief of the position is calculated by a 2-step update in which the first step must deal with ‘time update’, while the second performs the ‘measurement update’. Both steps can be expressed as:

(12) $$\bar{\text{bel}}(x_{t})=\int p(x_{t}|{\hat{x}}_{t},x_{t-1})\text{bel}(x_{t-1})dx_{t-1}$$

(23) $$\text{bel}(x_{t})=\eta p(z_{t}|x_{t})\bar{\text{bel}}(x_{t})$$

In the same way as MCL, Kalman filters can work with an on-board camera as the main perceptive sensor. Consequently, these filters can also be established as a functional localization system for general purposes, and specifically, RoboCup. Most approaches used in SPL are centered on multi-modal variations of the UKF and these demonstrate better results than the original implementation of UKF. Measurement functions must be previously linearized in order to obtain the Gaussian representation, and this usually leads to unsatisfactory results and a divergence between real and predicted positions.

The Austin Villa team (The University of Texas at Austin, Austin, TX, USA), winner of the 2012 RoboCup competition hosted in Mexico, used ‘Multi-modal 7-state UKF’ localization to obtain reliable estimations. As reported in [11], in a normal UKF, belief in a 7-dimensional space state involves seven distinct Gaussian representations; but the multi-modal filter only takes into account a single representation defined as the weighted sum of all seven Gaussians.

A general implementation for an *N* dimensional filter must be introduced as follows: given a concrete model *x(t)* with *N* Gaussian distributions, each representing the belief of an estimate estate *x_i_* with a covariance matrix *P_i_* and a weight *α_i_*, the final distribution of the model is expressed as:

(14) $$\text{bel}(x(t))=\sum_{i=1}^{n}\alpha_{i}\frac{1}{{\left(2\pi \right)}^{n/2}{\left|P_{i}\right|}^{1/2}}\beta$$

(15) $$\beta =e^{\left(-1/2{\left(x(t)-{\hat{x}}_{i}\right)}^{T}P_{i}^{-1}(x(t)-{\hat{x}}_{i})\right)}$$

The multi-modal approach was said to be in [12] a technique with a high computational cost because of the Gaussian distributions involved in the weighted sum. For this reason, a new method based on the general idea of the multi-modal filter was developed in an attempt to solve these issues. This new approach, introduced by Nao Devils in [12], is the ‘Multi-hypotheses UKF’. Deleting some of the terms in the Gaussian sum has been proposed to increase the efficiency of the filter. However, it must be pointed out that the discard method may lead to a loss of accuracy in estimation—as well as other problems.

As a new feature, a resampling step similar to that applied in the MCL has been added. In that step, the weighting updates are adjusted to discard the outlier values. This approach also adds new terms based on sensor measurements, independently of previous measurements. Low-weighted models were introduced enabling the filter to achieve a ‘sensor resetting state’ that will be explained in more detail below.

A comparison between UKF and MCL can be found in [13], which reviews the standard algorithms for both methods and discusses some simulation results. The main conclusion is that the MCL method is computationally worse than UKF. However, it must be pointed out that MCL still offers some relevant advantages: (a) MCL is more accurate in certain cases; and (b) MCL can deal efficiently with problems such as kidnapped robots. Moreover, some MCL implementations with a small number of particles can computationally perform in a similar way to the UKF, although performance may be compromised.

Localization systems for humanoid robots usually follow the path created by simpler mobile robots, such as wheeled robots. It is usually easy to find studies centered on new techniques and alternative methods that have not yet been transferred to the SPL frame. In [14] a variation of the UKF is introduced that implements a fuzzy logic adaptative system. As has been shown, the main problem in using KF is the inability to deal with non-linear systems because of the process and measurement noises; and the fact that sensor fusion is required. To improve localization accuracy, a fuzzy inference system has been designed to determine the bounds of that noise. In [15], a visual self-location evolutionary algorithm is presented that estimates robot location. For that task, several sets of individual positions (similar to particles) are characterized with a certain ‘health’ value obtained after the image analysis. Each of these individual positions is placed in the space based on the perceived information. A fine-grain search among all these positions is performed. This algorithm is specially designed for dealing with symmetries that work with current visual information or visual memory as is defined in the study. To validate this last method, two experiments are shown that involve the Nao robot in an environment that is different to RoboCup.

Finally, it must be emphasized that an efficient localization system must always include reliable and continuous information about the environment. Thus, the acquisition and processing of perceptual measurements is a key to success in the localization problem. In [16], several techniques and upgrades for producing faster game play and better localization in SPL are presented, and these include synchronized head movement, shared information between robots (similarly to the global ball model), and a path planning system.

## 3. Hidalgos Team Control System Architecture

**Figure 1.**
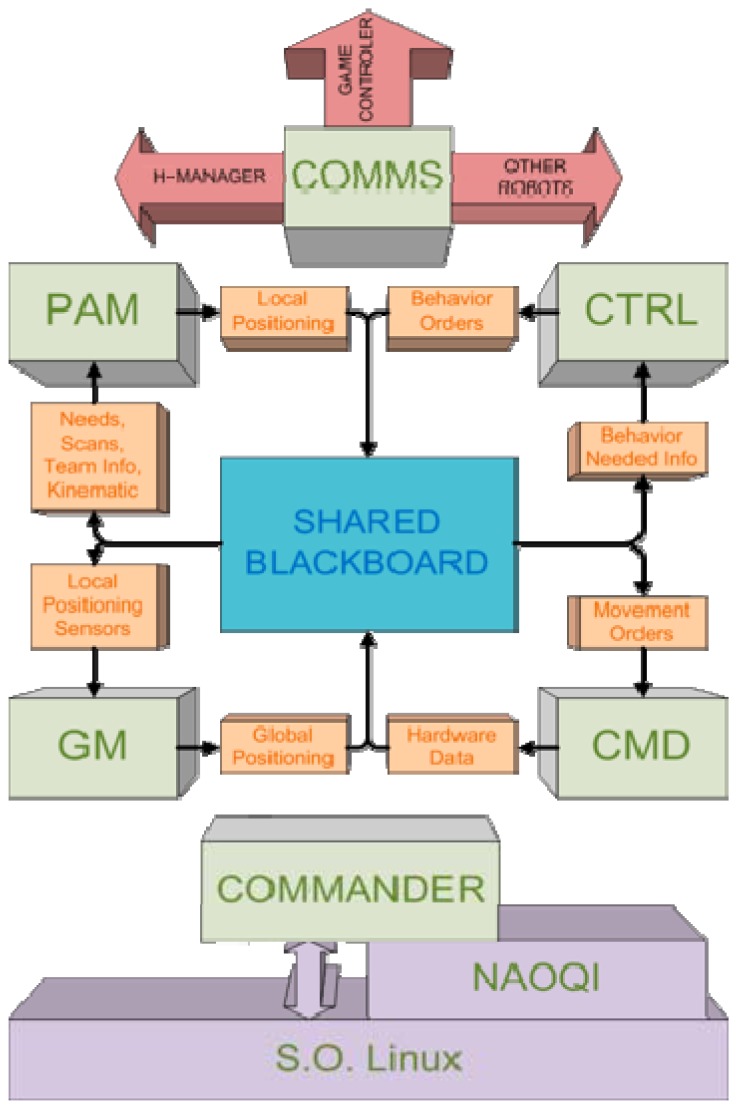
Hidalgos Team control system structure overview.

The Hidalgos Team control system is based on several SW modules encapsulated into libraries that are organized according to their function. The main modules of the system can be seen in Figure 1 and are termed:

- COMMS: Dedicated to communications between robots and game referee.
- PAM: Deals with the perceptual hardware for obtaining relevant environmental information.
- CTRL: Responsible of the correct execution of state machines and behaviors involved.
- GM: Intended for global modeling and position estimation.
- CMD: Manages access to hardware and system resources.

Obviously, to perform its tasks the CMD deals with the NAOqi middleware provided by Aldebaran Robotics and the OS (which in this case is an embedded Linux distribution). All the information that must be shared between distinct modules is exchanged through a shared blackboard structure.

Also, a distributed management and calibration tool, known as H-Manager, has been developed to adapt the robot configuration before the football match. Moreover, this H-Manager enables a quick switch between the selected modes and behaviors.

**Figure 2.**
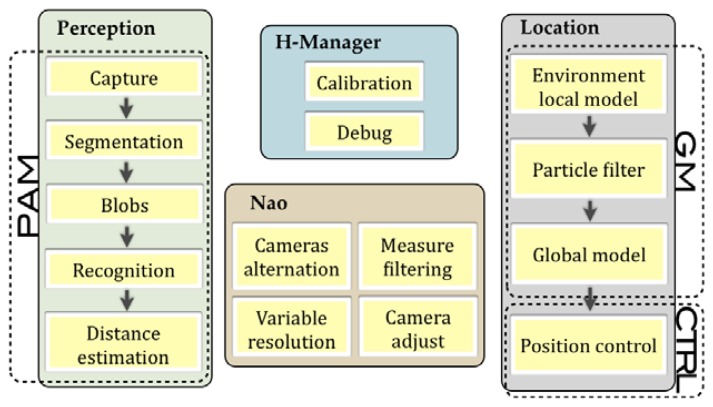
Diagram with the SW routines involved in location and positioning.

The proposed goals of location and positioning will be achieved by the combination of many steps. Some improvements in the perception system are needed to obtain a reliable localization. Several modules of the Hidalgos architecture may be combined to encourage an optimal flux—from the acquisition of the information needed by the system to the previous data treatment involved and described in Figure 2.

## 4. Perception System in the Hidalgos Team

As previously discussed, every type of localization system must have sensorial information about robot surroundings to determine the current position. This information will affect the performance of the localization system. Features in the game field are the main sources of information. Each spotted feature is usually characterized by a distinctive color, shape, and position. The issues related to the recognition of these characteristics will be detailed in this section. The sensor adjustments required for guaranteeing a reliable distance estimation and the auxiliary tool for obtaining the camera settings will also be detailed. In each case, the related problems and the adopted solutions are described.

### 4.1. The Nao Vision Sensor

**Figure 3.**
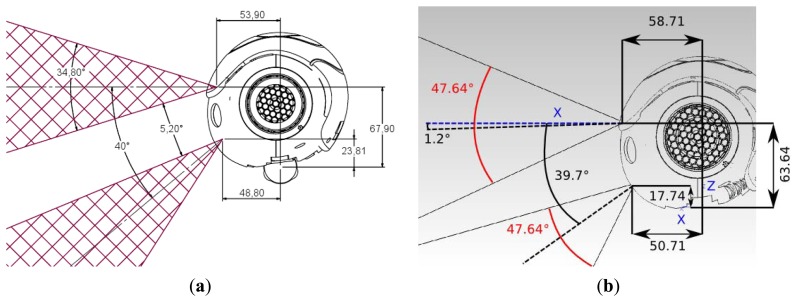
Camera placement in a Nao robot. Side views of the head camera layout. Note the field of view (FOV) of each camera: (**a**) Nao V3, (**b**) Nao V4.

Nao robots have two cameras on the head as depicted in the Figure 3. The two cameras have CMOS sensors that provide VGA images at a maximum of 30 fps. Both cameras have the same field of view (FOV) angle, which may vary depending on the version, as well as the same offset between the centers of the visual beams.

These differences can be appreciated by examining Figure 3. The robot head has been assembled on the body through a neck formed of two servomotors, which are responsible of the pitch and yaw movements respectively. These movements allow the robot to perform a visual scan to recognize objects in a large range defined as the frontal side of a sphere bounded by the configuration of the cameras and servos. These movements give Nao the capacity to perform visual servoing for tracking objects such as the soccer ball and to keep it in the FOV. A revision of the visual servoing procedures can be found in [17].

### 4.2. Image Capture

To perform better localization and more efficient game playing, the capture method has been improved by adding a camera management system to the Hidalgos project. Its main function is to provide an optimal use of both cameras by enabling the correct camera for each instant.

**Figure 4.**
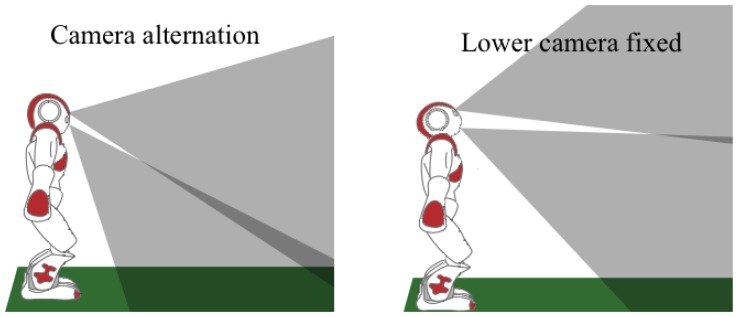
Different situations and camera modes: in the left picture, image information from both cameras is required, while in the right picture, only the lower camera is required.

For this purpose, three operating modes are introduced: upper camera fixed; lower camera fixed; and camera alternation. The first mode (upper camera fixed) is usually the least used mode due to its activation conditions and its effective range limitations. This mode will only be used in those cases where the ball or other relevant object is situated several meters from the robot and it must be tracked. The second mode (lower camera fixed), in contrast with the previous mode, is the mode most often used during the match. This mode must be active when the robot tracks an object situated near the robot. The third mode (camera alternation) is activated when a scan of the environment must be made and the limits of the game field do not appear in the FOV of the lower camera, as illustrated in Figure 4. This third mode is also used when a shot-to-goal must be made and the ball cannot be framed with the goal in the same picture. By using this alternation, a considerable amount of unnecessary head movements are saved and more information is obtained per cycle.

**Figure 5.**
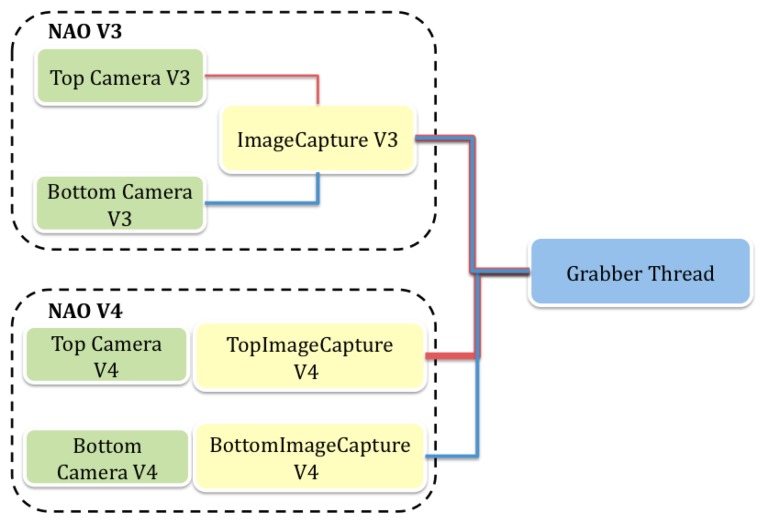
The camera management system offers support for camera activation and switching—depending on each robot version.

Although both versions (V4 and V3) of the Nao robot have two cameras, the hardware configurations vary. The newer V4 has two separate ports for each camera, but in the V3 all the cameras use a single port and a switch is used to enable each. Taking this into account, some relevant structures have been developed to independently perform image acquisition on both cameras. The grabber thread must apply the appropriate method according to the version of the robot that is running the code, as shown in Figure 5.

### 4.3. Segmentation

**Figure 6.**
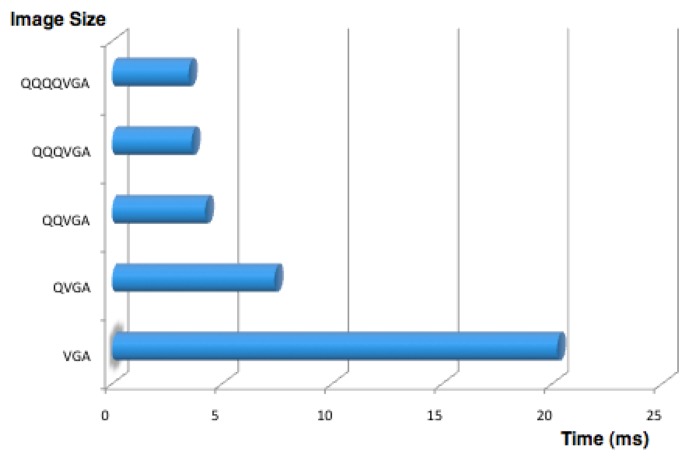
Segmentation times vs. different image resolutions.

Once a frame is captured, the contained information must be extracted. The first step is to perform the segmentation process by colors. A look up table (LUT) is used for this purpose, which has previously been configured using the H-Manager application for the definition of the range of values that match each color. This process has a high computational cost, as it must examine each image pixel and determine its color. To lower these costs, a subsampling method known as scanlines was implemented as described in [18]. This technique avoids processing the whole image by skipping certain pixels, and so produces the same cost as would be obtained by processing a low-resolution version of the picture. Thus, the number of skipped pixels in this process is strictly related with the computational costs as shown in Figure 6. However, skipping pixels increases the difficulty of finding a feature in the image. If the number of ignored pixels is too high, some information may be missed. To preventing this problem arising, a system for managing the subsampling method was implemented.

The vision system must select a suitable resolution every time. The current position of the head and active camera must be taken into account. Thus, when the robot is looking at its own feet the lowest resolution can be used, because all the spotted objects will fill a large part of the image and so enable a remarkably large amount of pixels in the image to be skipped. In other cases, such as when the top camera is being used, almost the whole image must be processed because some objects such as the ball will only represent a few pixels on the image and skipping some can induce it to overlook the ball. Nevertheless, in particular cases, such as when the robot is looking for big objects like the goal, the resolution can be slightly lower without changing the results.

### 4.4. Blob Forming and Object Recognition

**Figure 7.**
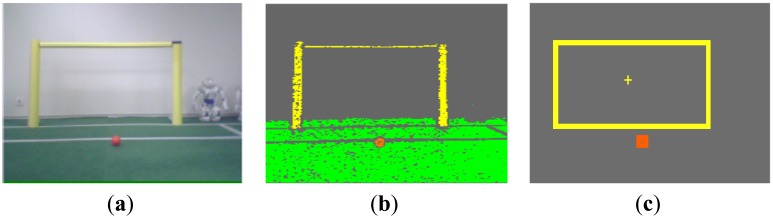
Results from image acquisition and processing: (**a**) Original picture; (**b**) Segmented image; (**c**) Bounding boxes of the goal and ball.

Starting from the segmented picture, all the contiguous regions of the same color must be assembled, forming a blob that will potentially represent a game feature. For this purpose, the seed region growing (SRG) technique [19] is used in our approach. Figure 7 shows the complete sequence followed in a real image.

By providing the spotted blobs, the recognition system can determine which corresponds with a game object and (if it were the case) what type of object they represent. Blob classification is performed by analyzing characteristics of the blobs such as their size, position from the horizon, and other parameters that can be configured using the H-Manager application.

### 4.5. Distance Estimation

One of the most important measurements that can be extracted from the blob is the distance between the robot and the feature. Nevertheless, the process to obtain this value from the image is not trivial. Thus, a distance estimation procedure must be implemented. In this process, several camera factors that relate the real 3D world objects with the plain 2D figures found on the image are involved.

**Figure 8.**
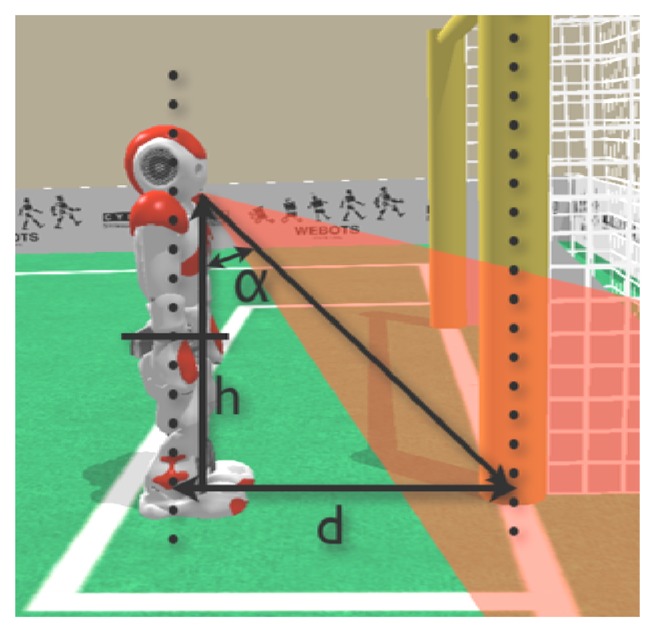
A simple geometrical calculus can provide the distance estimation of the feature of interest.

For computing the correct relations, a two-step adjustment process of the camera has been proposed in this paper. The first step deals with the intrinsic adjustment to compensate for lens distortion, while the second step performs an extrinsic adjustment based on the position of the camera. Finally, by a simple triangulation involving the real angle between the camera and the vertical axis of the robot and its height, the real distance can be estimated as illustrated in Figure 8.

**Figure 9.**
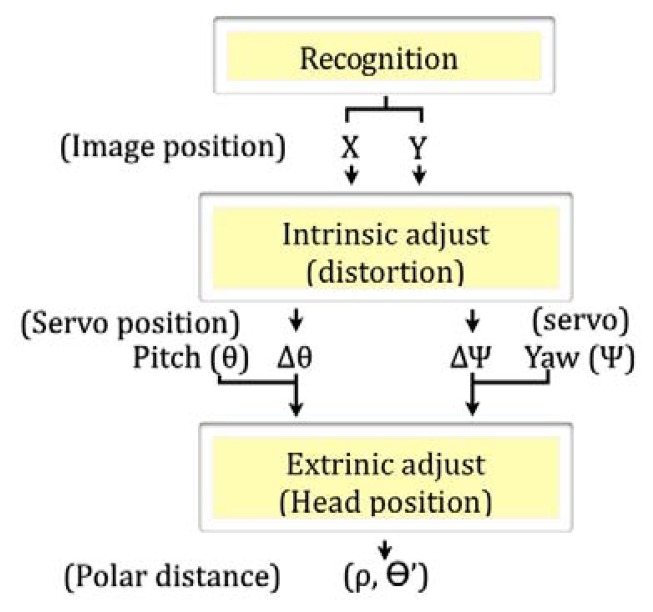
Diagram for object-to-robot distance estimation.

Once the camera has been adjusted through this process, the distance between the robot and the object of interest can be obtained, expressed in its polar form, and starting from the visual information. The whole process is represented step by step in Figure 9.

## 5. Camera Settings and Error Compensations

As discussed in the previous section, the camera settings are the key for success in vision-based distance estimation. For that reason, it is necessary to obtain the sensor model and the parameters that enable us to perform the camera adjustment. There are many studies focused on camera-based distance estimation and camera adjustments. A clear example can be found in [20].

### 5.1. Intrinsic Adjustment

Intrinsic parameters are required to compensate for the distortion effect on the captured image produced by the curvature of the camera lenses. Although there are several relevant methods such as those proposed by [21] or Tsai [22], we selected the method based on the Zhang [23] procedure, as suggested in [24]. By extrapolating the previous conclusions obtained in Section 4.5, we can assume that a relationship can be found between the number of pixels in the image, the angle of separation between them, and the real position of the camera in the 3D world.

By using pixel-angle conversion it is possible to estimate the correct distance to non-centered features. The aperture of the lens, in both horizontal and vertical axis, and the image resolution, characterize this conversion. Unlike the intrinsic case, this correction must be applied separately from the top and bottom camera, as there are no two identical lenses.

**Figure 10.**
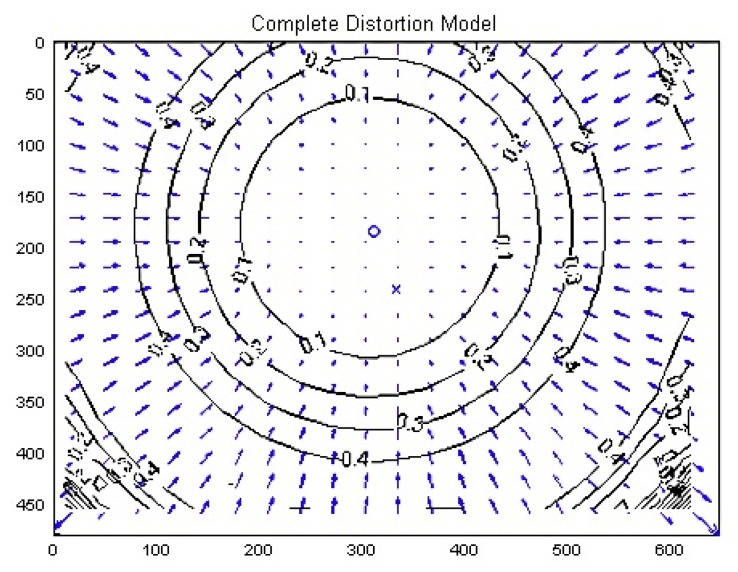
Example of radial distortion of the Nao camera lens: the coefficients marked on each circle represent the factor of distortion induced in each pixel located in that area (units are in pixels).

It must be also pointed out that lenses introduce additional non-linear distortion. There are several types of distortion, which can be defined as barrel or pin-cushion. The shape of the distortion in the image may vary as defined in [23]. After exhaustive analysis of the Nao camera, Figure 10 shows an example of the radial distortion of the lens.

The correction must be made along each axis if the lens does not have to be symmetrical. In this particular case, the ‘estimating radial distortion by alternation’ method was applied as described in [23] in the ‘dealing with distortion’ chapter. This method involves the use of three parameters *k_i_* for each axis obtained from the following Equation (16):

(16) $$\left[\begin{array}{c} \begin{array}{ccc} \left(u-u_{0})(x^{2}+y^{2}\right) & (u-u_{0}){\left(x^{2}+y^{2}\right)}^{2} & (u-u_{0}){\left(x^{2}+y^{2}\right)}^{4} \end{array}\\ \begin{array}{ccc} \left(v-v_{0})(x^{2}+y^{2}\right) & (v-v_{0}){\left(x^{2}+y^{2}\right)}^{2} & (v-v_{0}){\left(x^{2}+y^{2}\right)}^{4} \end{array} \end{array}\right]\;\left[\begin{array}{c} k_{1}\\ k_{2}\\ k_{3} \end{array}\right]=\left[\begin{array}{c} \overset{⌣}{u}-u\\ \overset{⌣}{v}-v \end{array}\right]$$

New capability for dealing with the distortion has been implemented in the system—depending on the radius from the image center to the pixel of interest.

### 5.2. Extrinsic Adjust

The extrinsic parameters of the camera describe the coordinate transformation between the robot and the object represented by a given point of reference in an undistorted image. These parameters are strictly dependent on camera position as seen in [20]. The distance measured is directly related to the head position that relays the configuration of the servo-motors at this time. A study of the characteristic profile of each servo involved on the camera movement must be made. The profile of the servo positions has been introduced as the main factor responsible for the equivalence between an image point and its corresponding point in the 3D world. In other words, this is the main reason why a full study of the behavior of the motors will lead to an accurate distance estimation.

It must also be taken into account that when the camera plane and the ground plane form a near π/2 angle the resolution on the measurements decreases drastically. Thus, this situation must be avoided during operation of the robot, and the head pitch angle must be carefully adjusted. This adjustment will rely in the NAOqi's kinematic system, which can provide a mostly reliable measurement of the camera height; while the value of its yaw angle can be modeled with a fixed offset.

**Figure 11.**
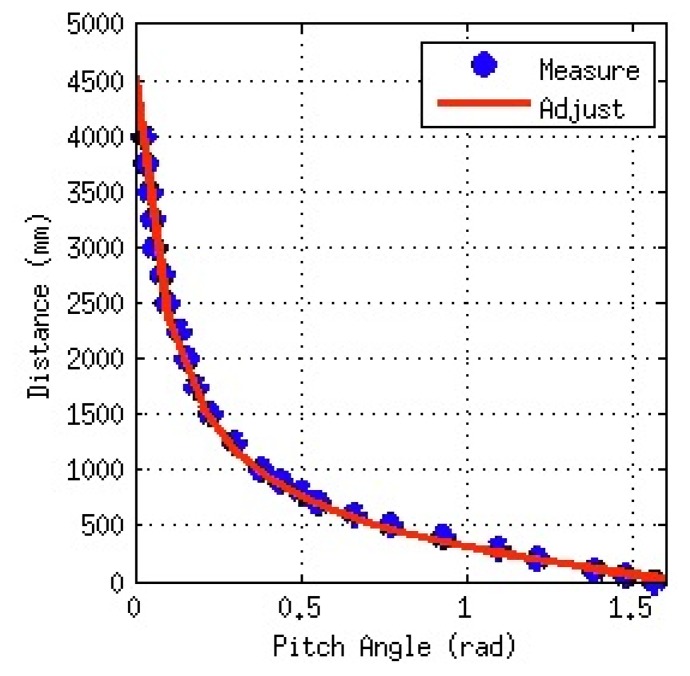
Example of polynomial adjustment (based on empirical measures) that establishes the relationship between head angles and distance-to-features centered in an image.

By making an empirical test, the profile of the camera pitch movement can be obtained for every robot, associating the central point of the image with its corresponding distance in the real world. Figure 11 shows a polynomial function that can be used to adjust the shape of the empirical measurements, giving us the capability of making a straightforward conversion from angle to distance. This procedure can be applied for both cameras, taking into account that they use the same motor, and consequently, present the same adjustment, but with the offset between camera positions.

### 5.3. Auto-Adjust Procedure

After both extrinsic and intrinsic parameters have been obtained, a reliable estimation can be performed. However, it must be taken into consideration the fact of that each robot is likely to show some differences in the values of the configuration parameters. Moreover, these values may be modified over time due to mechanical damage (falls or knocks). This is the main reason we designed an auto-adjusting system—so that each robot can obtain its own parameters by executing an autonomous procedure.

Focusing on this idea, an OpenCV based application has been developed to obtain these parameters and store them on an XML file located in the robot memory; enabling the configuration parameters to be loaded each time the robot is restarted. To adjust both (extrinsic and intrinsic) types of parameters, the robot must be placed in a known position and once there, it must recognize some characteristic features of the game field (such as corners or line crossings) using the OpenCV application.

The auto-adjust system is designed as a new functionality offered by the previously mentioned H-Manager tool. The developed algorithms are not running in the robot hardware, instead of this the application applies for a picture from the robot and the subsequent image processing is carried out using the H-Manager. The application then calculates the new position for each head servo, and sends the information to the robot again. Thanks to this mechanism, the adjusting process can be supervised by the computer; working quickly in a distributed and supervised manner.

First of all, the extrinsic parameters will be obtained by making the servomotors of the camera move to locate the lines of the game field, whose distance to the robot is known because the robot's position is known. The obtained (distance, pitch angle) pairs of values measured during the camera movement are used to obtain the parameters of the polynomial adjust, as shown in Figure 11.

To find the robot position that offers the best calibration through the lines, an optimization method has been developed that provides the required location by evaluating all the potential positions.

The described calibration procedure is applied on the head bottom camera, and taking into account that both head cameras move in common (always having a fixed offset between them) the adjusted polynomial can also be used for the upper camera by accounting for this offset between cameras. This offset between cameras can be measured for better performance by finding the same point with both cameras and computing the difference between the reached positions.

**Figure 12.**
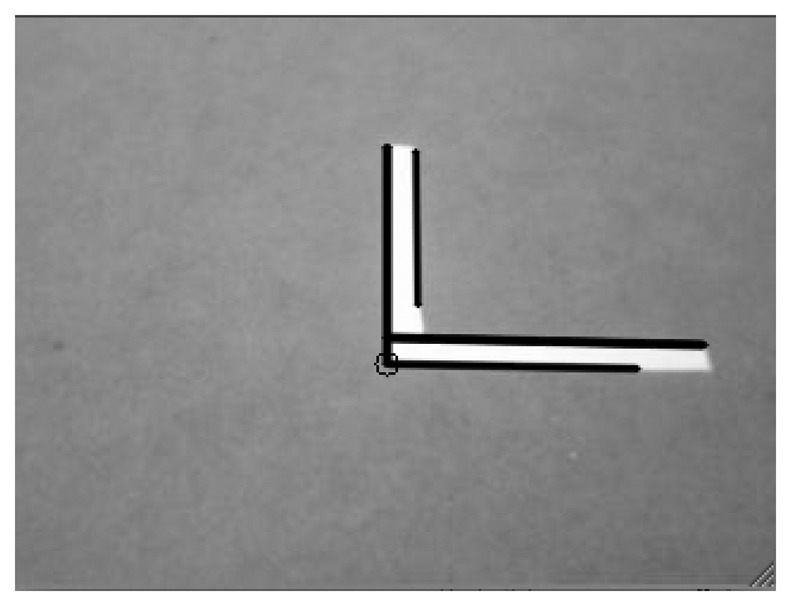
L-shape recognized on an image (a corner of the game field). This pattern helps us to obtain camera settings.

The next step in the auto-adjust procedure is to obtain the intrinsic parameters to compensate for lens distortion. For this purpose we have chosen to use a game field feature that is easy to locate and has a well-defined point: the corner. Thus, the camera is moved to locate the corner point in the center of the image, which is supposed to be unaffected by any kind of radial distortion. Once this point is reached, the camera must be moved to find the same feature but from in a non-centered position. This operation is repeated several times, enabling several images to be captured that situate the same feature in new positions at different radii from the image center and covering all the value range. Equation (16) can then be applied to the experimental data, producing a distortion map similar to the one represented in Figure 10. During this intrinsic adjust, the image processing algorithm is charged with the task of finding the exact corner point from the L-shape of the image, as shown in the Figure 12.

### 5.4. Estimation Results

To evaluate the above described auto adjust procedures; an experiment was conducted to compare the errors in distance estimation without auto adjust by taking the default parameter values with the errors produced under the same circumstances; but after applying the auto adjust procedures. As indicated; one of our goals was to achieve the capability of measuring distances between the robot position and a given feature.

**Figure 13.**
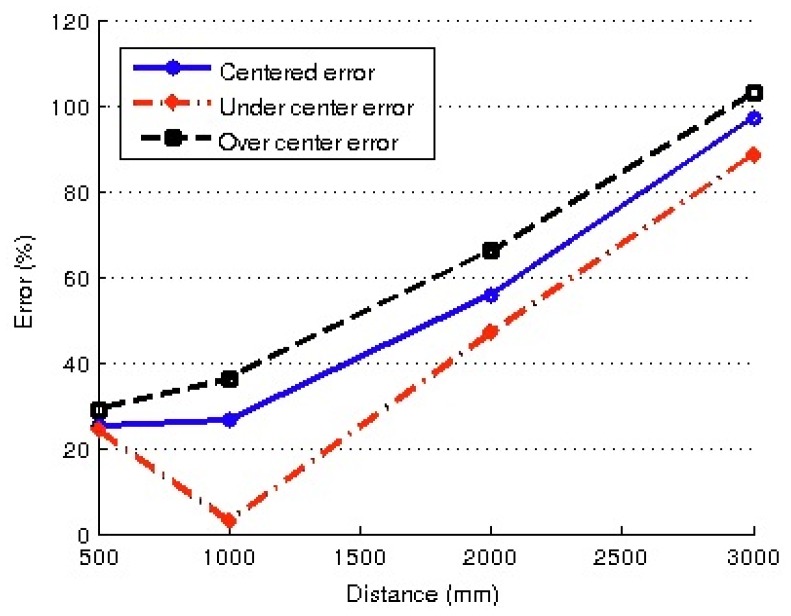
Errors in distance measurement when tested without adjustments.

The experiments were designed as follows: firstly, the robot was manually placed at several distances from the same object, ranging between 0.5 m and 3.0 m, and the distance was estimated using simple triangulation, as indicated in Figure 8. For each position, the distance was measured by moving the head to locate the feature of interest in three positions in the image: (a) A feature located in the center of the image (centered); (b) A feature located in the lower half of the image (under centered); and (c) A feature located in the upper half of the image (over centered). These three measuring approaches were chosen to deal with different coefficients of distortion. The errors obtained for the three centering approaches are shown in Figure 13.

**Figure 14.**
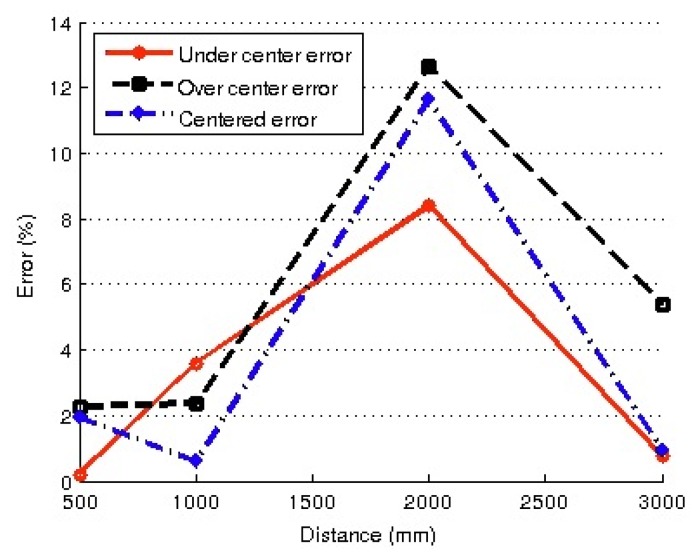
Errors in distance measurement when tested with adjusted parameters.

In a second phase of the experiments, the robot was re-positioned in the same locations as the previous experiment, but in this case, the distances using the same object were measured by taking into account the adjusted values of parameters, following the auto-adjust procedure above described. The new set of errors obtained in the distance estimation is shown in Figure 14.

In view of the results shown in Figures 13 and 14, it is clear that the set of results obtained using the auto-adjust procedure was far more accurate in distance estimation in all the cases: ranging from 2% for short distances and 13% for distances of two meters (the worst case). Unadjusted results yield errors of 20% for distances of 0.5 m, increasing with distance values to 100% for 3 m.

Obviously, unadjusted estimation is not a suitable method for distance estimation, because world modeling requires a more precise localization system. Fortunately, the accuracy obtained with adjusted parameters seems adequate for world modeling purposes using the proposed particle filter.

## 6. Environment Data Processing and Local Modeling

Once the robot can estimate the distance to objects detected in a captured image, relevant information about the main game field features can be obtained. This information will be useful to construct the surrounding environment. By processing this data we obtain a local model of the game space.

### 6.1. Landmarks

Some elements of the game field in the SPL can be used for information about the robot position. However, there is no single distinctive feature that can be used as a single solution. The model of the local surroundings can help to reduce the options. Landmarks are defined as the field features to be modeled. Currently, the main landmarks used are: the goals, the borderlines of the game field, and the information shared by others robots of the same team. Other landmarks have been discarded because the increase in computational cost was not compensated with a relevant improvement in position estimation. However, future developments based on newer Nao versions (equipped with more powerful CPUs) open new possibilities for improvement.

**Figure 15.**
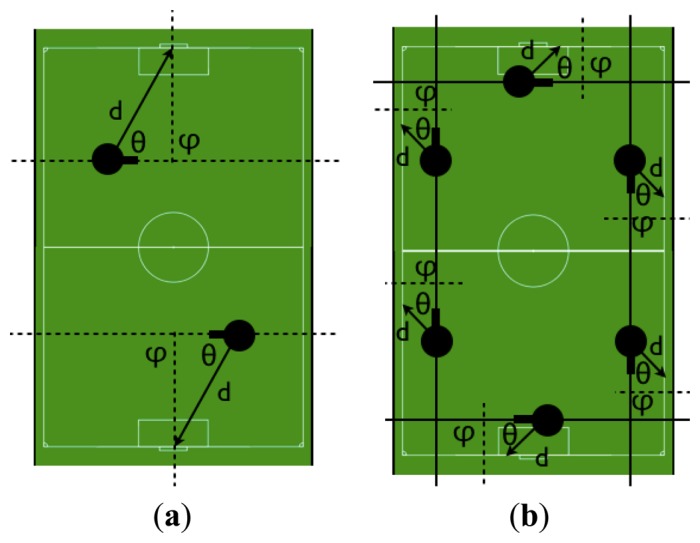
Possible robot positions (d, θ, φ) deduced by a given landmark detection: (**a**) goal information; (**b**) borderline information.

The goal position usually generates sufficient information to determine the position of the robot in the field. This measure has been defined as the relative distance from the robot current position to the goal and the inclination angle between the robot orientation and the imaginary axis passing through the crossbar expressed in the global system. By considering this information there are only two possible situations on the field, as shown in Figure 15a, but usually the result of previous estimations helps to nullify one of the options and resolving any uncertainty about the robot position.

White borderlines also give information that help locate the robot position in a point situated on the parallel line separated from the border at the measured distance, with an orientation based on line inclination. Figure 15b shows how this information only provides a rough approximation of the real position, but offers good information if the robot cannot see the goal and so prevents the particles from being scattered around the field.

Although landmark detection offers an acceptable localization, this is not enough to solve the problems caused by the game field symmetry, given that without previous information there is no way to discern which midfield the robot playing in—no matter how good the sensed information. By inputting the shared information (using communication between players) from robots with a reliable position, the robot can reinforce its own information and more quickly converge to a single position by discarding an incoherent symmetric position.

### 6.2. Landmark Modeling Filter

Distance information from each detected landmark is used to model the local surroundings as discussed before. As indicated previously, using the auto-adjust procedure already described can drastically reduce the errors in distance estimation, but it must be taken into consideration that the distance measurement tests have been made with a motionless robot. In real game situations, it is highly desirable that the robot be able to make dynamic distance measurements while walking.

Locomotion vibrations and bounces while walking will affect the quality of the estimation by adding noise—which prevents a stable local model being obtained. A filter is proposed to reduce noise and enhance the dynamic distance estimations.

Induced noise appears as a high frequency signal added to the real values. Using a simple mean filter could mitigate this problem. After analysis of the usual noise characteristics, we have designed a simple moving average filter that obtains the mean of the last 50 distance samples. This type of filter is optimal for removing the noise and can be implemented using a fast recursive approach [25]. The designed filter will take the last 50 samples to obtain the filtered value, assuming a quasi-constant acquisition rate of 55 samples/s. The time window is about 0.9 s. The filter size has been chosen as a result of numerous tests showing that the best cost/performance ratio was 50 samples per window. However, if the robot loses sight of a certain feature, the filter stops and resets all the previous values to avoid using invalid results.

One of the main problems associated with this kind of filter is the delay produced in the resultant value. To cancel the effect of this delay, a prediction step has been added to the filter. In this step, the trend of the previous filtered values is analyzed, making an estimation based on the current trend to predict the real value instead of the delayed value. Although the predicted result improves the time response, it may produce some added noise. Bearing this in mind, another study has been made to determine the level of prediction that offers the best balance between temporal and noise reduction performance.

### 6.3. Data Integration and Local Modeling Results

Once each feature detected in the game field has been analyzed, the system fuses all the information to establish a local model. This model represents the position of the features detected at a given time, deleting all the information relative to previously detected elements that have been lost from sight. This action avoids the actualization of local data with the odometrical information and prevents the system working with false values.

**Figure 16.**
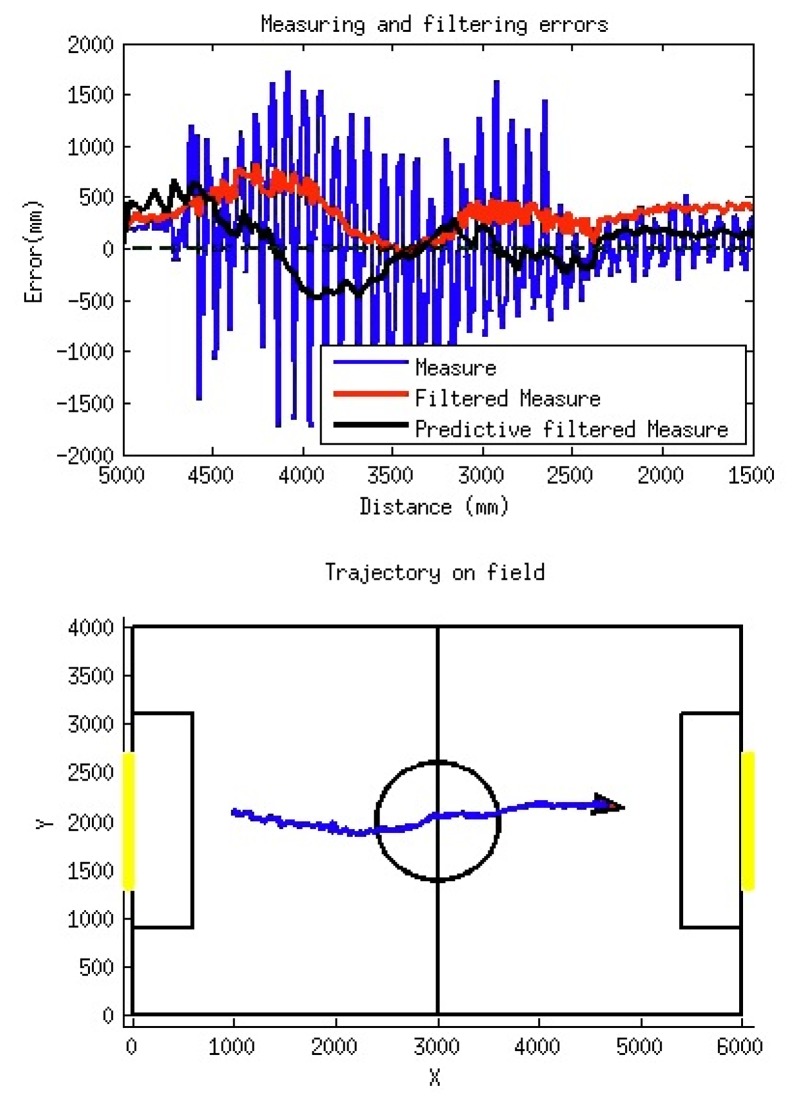
Measured distance from robot to right goal. Lower plot: robot trajectory during walk. Upper plot: obtained errors in distance estimation during this walk.

Figure 16 shows a simple experiment in which the robot is approaching the goal. During this test the real trajectory described by the robot is stored (obtained from a zenithal camera system) and the distance measured to the goal. This last value will be used for local modeling and will describe the information of the featured position relative to the situation of the robot. The lower 2D plot in Figure 16 shows the real trajectory of the robot during its displacement from left to right.

The upper part of the Figure 16 shows the distance errors (in millimeters) obtained during this displacement as a function of the real distance to the right goal. In this plot, three different sets of data have been represented: (a) measured raw data (blue, very noisy); (b) data after filtering (red); and (c) predicted data from filtered values (black).

Figure 16 illustrates how the quality of the measured distance has been notably enhanced by means of the simple filtering procedure described. In the non-filtered data (in blue), the effect of the walking bounces on the readings is evident. Also, the filtered set (in red) presents a very reduced amount of noise, but a biased response can be also appreciated, giving a positive mean error of about 250 mm (the distance is over-estimated due to the time delay introduced by the filter).

Finally, the predicted data set (in black) shows a balanced behavior, as the mean error is near to zero, while the amount of noise is also very reduced—with a maximum value during the robot walk of 600 mm for distances above 4 m. As a conclusion, the proposed filter for the distance measurements enables us to obtain errors in distance estimations within tolerable margins for local modeling purposes.

## 7. Localization System: Algorithm Description and Results

### 7.1. Auto-Localization System and Global Modeling

This section describes the algorithm that has been developed for robot self-localization. As previously indicated, we have used a modified version of the particle filter, taking into account some features of the augmented filters and techniques inspired by the UKF already used by other RoboCup teams. Our implementation corresponds with the following description.

Given a distribution of a set of *X* particles where each element is defined as:

(17) $$X_{k}=[x_{1‥n}]=\left[\begin{array}{c} {\left(x,y\right)}_{1‥n}\\ \varphi_{1‥n} \end{array}\right]$$

For every filter iteration, the values of the distribution are updated according the translation and rotation movements carried out by the robot, which are expressed as an increment in the odometrical values given by NAOqi. This action will be represented as action *a_k_*; and a noise *v_k_* will be added to model the odometrical error affected by the reliability coefficient *R* which will be introduced below:

(18) $$X_{k}=f(X_{k-1},\alpha_{k})+v_{k}R$$

A new distribution *Z* is defined as the probability of making a correct estimation of all the features in the field, in this case the goal and the ball. Therefore, each value of probability *z_i_* is associated with the probability of having a successful estimation *S* using the particle *x_i_* included in the distribution.

(19) $$Z_{K}=[z_{1‥n}]=\left[\begin{array}{c} p^{{\text{Goal}}_{1‥n}}\\ p^{{\text{Lines}}_{1‥n}} \end{array}\right]$$

(20) $$z_{i}=p(S|xi)$$

The values of *Z_k_* are obtained as a function of the elements in *X_k_*, the previous estimation *E_k-1_*, the perceptual observations at time *η_k_*, and the well-known model of the game field *M*:

(21) $$Z_{k}=f(X_{k},E_{k-1},\eta_{k},M)$$

This procedure belongs to a ‘coherence system’ that tries to find the common elements from the global information perceived by the robot.

Once the *Z_k_* distribution is available, the quality of the estimation can be obtained by computing the probability of having a successful estimation using each sample on the *X_k_* distribution:

(22) $$p(x_{i}|S)=\frac{p(x_{i})p(S|x_{i})}{\sum_{x=1}^{x=n}\left(p(x_{x})p(S|x_{x})\right)}$$

Consequently:

(23) $$p(x_{i}|S)=\frac{nz_{i}}{\overset{x=n}{\underset{x=1}{\text{∑}}}z_{x}}$$

The set of probabilities of success will determine the value of the reliability *R* as shown below:

(24) $$R=\overset{i=n}{\underset{i=1}{\text{∑}}}\frac{p(x_{i}|S)}{n}$$

This coefficient will determine the accuracy of the estimation of the distribution *X_k_*. It may also affect the amplitude of the odometrical noise by adding more dispersion to the actualization of the particle positions when required.

The next step is to obtain *x_ref_*, which is defined as the ideal member for the distribution *X_k_* based on the perceptual observations *n*, the model a priori *X*, and the previous estimation. In this way, *x_ref_* represents the theoretical position that makes the measured observations adjust to the known model of the game field:

(25) $$x_{\text{ref}}=f(E_{k-1},\eta_{k},M)$$

This new element will take part in a new distribution *X'* defined as:

(26) $$X_{k}^{'}=X_{k}\cup x_{\text{ref}}$$

The proposed estimation *x_ref_* must always guarantee that:

(27) $$\begin{array}{l} z_{\text{ref}}=f(x_{\text{ref}},E_{k-1},\eta_{k},M)=1=p(S|x_{\text{ref}})\\ p(S|x_{\text{ref}})=1\to p(x_{\text{ref}}|S)\geq p(x_{i}|S)\forall x_{i}\in X_{k} \end{array}$$

The obtained reference particle is the most adequate location according to the sensorial information, and is obtained through the application of the ‘coherence system’.

Before proceeding with the resampling phase of the filter it is necessary to assign respective weights to each particle. These weights are represented by the distribution *W*:

(28) $$W_{k}=[w_{1‥n}]$$

The value of each weight is obtained as the normalization of every probability of success between the minimum and the maximum value in the estimation, which corresponds to the *x_ref_* probability.

(29) $$w_{i}=\frac{p(x_{i}|S)-\min(p(x_{x}|S)\forall x_{x}\in X)}{p(x_{\text{ref}}|S)-\min(p(x_{x}|S)\forall x_{x}\in X)}$$

For this implementation, the number of resampled particles is dynamically modified according to the *W_threshold_* value, which determines the minimum particle weight to remain for the next iteration of the filter; otherwise it will be surrogated with a copy of *x_ref_*. This *W_threshold_* is obtained as a function of the reliability coefficient *R* and the weight variance *s*.

(30) $$W_{\text{thresshold}}=f(s,R)$$

The variance *s* defined as:

(31) $$s_{n}^{2}=\frac{1}{n-1}\sum_{i=1}^{n}{\left(W_{i}-\bar{W}\right)}^{2}$$

Finally, the estimation of the pose *E_k_* is obtained by performing a weighted mean of the components of the distribution *X'_k_* that offers the maximum weight and the reference particle *x_ref_*.

(32) $$E_{k}=\frac{x_{\text{ref}}+\sum X'W}{\sum W}$$

This procedure is reflected in Algorithm 1 as follows:

**Algorithm 1**: Modified particle filter
 **for** i=1 **to** N **do**  x(i) <- prediction(x(i), a,R)  z(i) <- probabilityCoherence(x(i),n,M, E) **end** **for** i=1 **to** N **do**  pS(i) <- calcProbability(x, z)  R <- actualizeReliabity(probS(i))  minP <- isMin(probS(i), minP)  maxP <- isMax(probS(i), maxP) **end** (x_ref, p_ref)<- referenceCoherence(E,n,M) W<- scaleWeights(pS, p_ref, minP, maxP) s <-calcVariance(W) *(_x,_w)<-addAndSort(x,x*ref,w) w_thresshold <- findThreshold(W,s) **while** _w(i) < wThreshold **do**  *x(i)<- resample(x*_ref)  i++; **end** E<-fuseBestParticles(_x)


The main difference between the presented implementation of the particle filter and others is that the weights being assigned are not based on the probability of each particle being in the supposed location, but rather on the closeness of each particle to the calculated reference particle. The sensor reset is dependent on the size of the best error. If the error value is low, most of the particle values must gradually reach the real position by updating their position to locations near the reference position—which produces the best error situation. If the best particle shows incoherence between sensorial measurements, the threshold value will be increased. This will force the filter to generate new random particles and enable convergence to the real position.

All these new contributions produce a filter characterized by a fast response to each potential situation. After several empirical tests, this solution has been shown to perform with a small number of particles in comparison with other implementations. As will be discussed in the results section, the designed filter can perform satisfactorily when starting from only 20 particles.

### 7.2. Positioning System

Once the position has been estimated, the implementation of a position control is trivial. Nevertheless, the challenge is to decide which position needs to be reached by every robot. This control only takes part in the game at kick-off, the start of the second half, or after a goal. The positions are assigned according to the designated player positions. Each robot has a position, which determines its role in the team, and it is considered satisfactory when the robot approximately reaches the indicated position, without invading forbidden areas (such as the opposite half or the goalkeeper area). The position system structures can be used as the core of future works, for example, in the implementation of complex trajectories.

### 7.3. Results

#### 7.3.1. Validating the System

For a validation it is necessary to acquire both the estimated and real positions. In the first case, the robot has the task of writing the estimated position in a file at the same time that it actualizes its global position on the GM module. In the second case, the complexity increases because there is no direct way to determine the real position. For this reason, an auxiliary platform has been developed to acquire this value.

**Figure 17.**
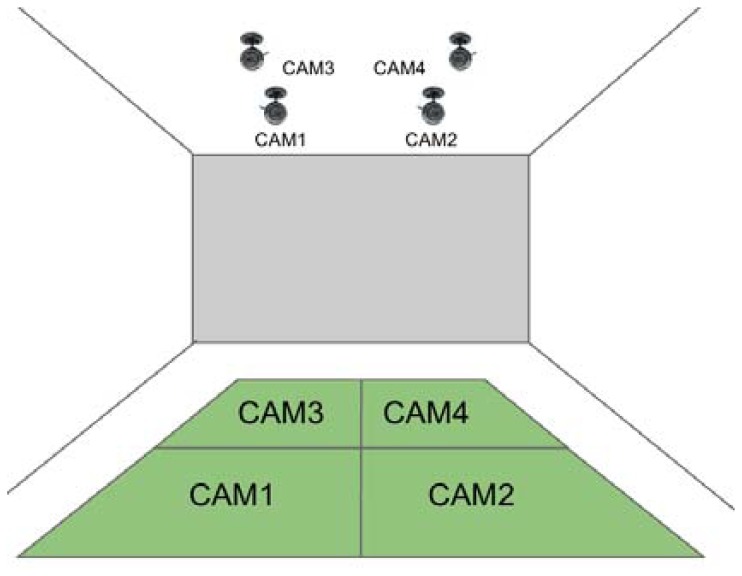
Graphical overview of the auxiliary platform for measuring the real position of robots. Note the four cameras distributed on the ceiling of the game field.

This platform consists of four webcams on the ceiling of the stadium (the number of cameras conditioned by physical restrictions—such as the low height of the ceiling or field of view). Each webcam detects each robot that enters its corresponding zone of the game field, as shown in Figure 17.

**Figure 18.**
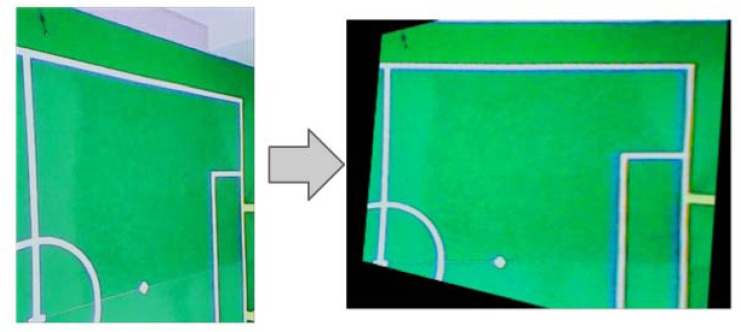
Perspective correction of the captured images. Left: original image, Right: image after homography.

Obviously, this camera arrangement will produce perspective distortion due of camera positions, but a homography procedure shown in Figure 18 is performed to obtain a zenithal perspective.

**Figure 19.**
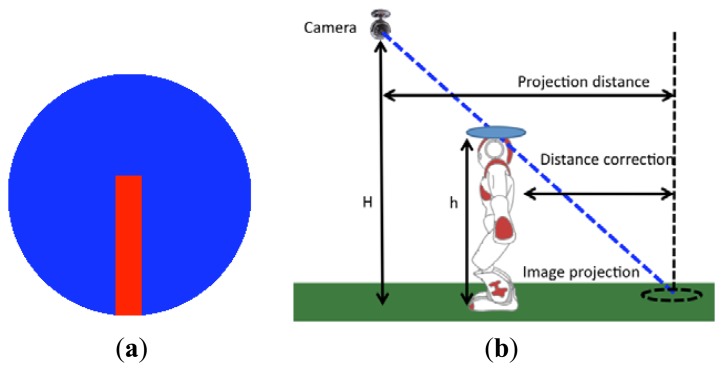
(**a**) Circular disk placed on the head of the robot for ceiling camera detection. (**b**) Correction of the perspective effect.

A computer vision algorithm has been developed that can detect each robot by searching on the image for a well-defined circular shape—as seen in Figure 19a. Once detected, the disk will determine the robot position and its orientation, after a correction of the viewing angle of the head of the robot (which produces a false projection on the ground). This adjustment is described in Figure 19b.

The heights of both the ceiling camera and the robot head are used to compute the over-measure produced (as shown in Figure 19b) and the distance correction is obtained.

**Figure 20.**
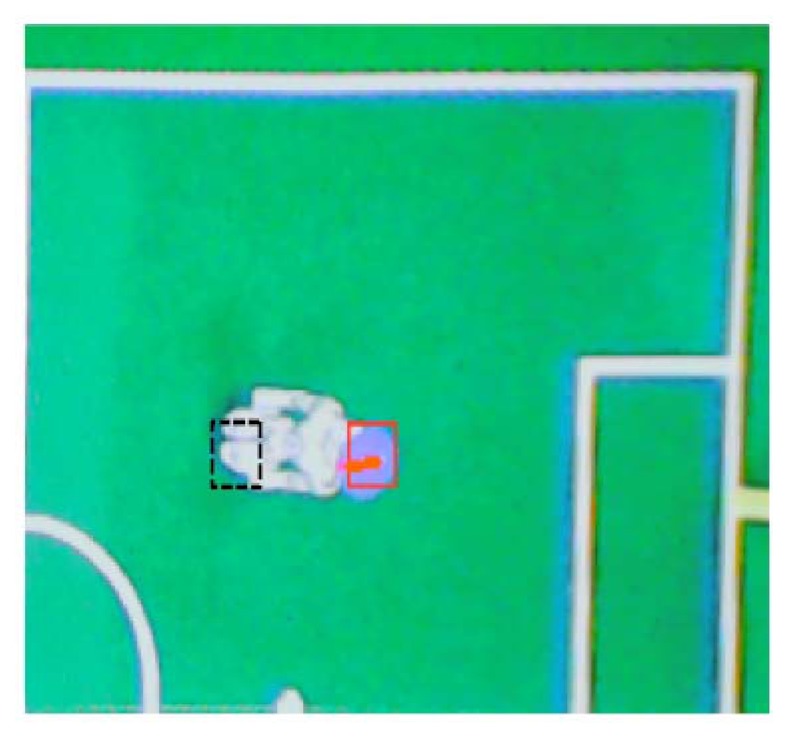
Recognized (red square) and corrected (black square) position of a robot in the image.

One example of a real situation is introduced in Figure 20. In that image, a robot position is detected by the recognition of the disk (red box). The corrected real position of the robot has been marked with a black box (corresponding with the feet positions).

The ceiling camera system works with four QVGA images due of limited bandwidth restrictions in the installation. These four images detect the robot on the game field. Because of low resolution and the limited configurable parameters of the cameras, the results suffer a positioning error that can vary from ±10 mm to ±100 mm and an orientation error from ± 5° ± 15° depending on the area.

#### 7.3.2. Localization and Positioning Results

To appreciate the improvements obtained with the proposed localization system a set of tests was carried out after selecting the number of particles to be used. As previously indicated, the reduced number of particles needed for the filter performance is one of the benefits of our proposed method. Therefore, we must determine the optimum number of particles. Several normal playing situations have been performed configuring the filter to work with different numbers of particles, storing in each case the computational cost of execution. Each execution was running for about five minutes, and the times were computed as the difference in time before and after the filter routine. The results obtained are shown in Table 1. For each selected number of particles it was determined if unstable situations due to the non-convergence of the algorithm occurred or not, and marking as *stable* or *unstable* the corresponding test.

As expected, the results in Table 1 show that a lower number of particles leads to slower execution times in a quasi-linear behavior. However, we must also be taken into account that for 10 and 15 particles, instability in execution was detected. Therefore, 20 particles is the minimum number of particles that guarantees stable behavior and a reduced execution time. For that reason, the following tests were performed using only 20 particles because it offers a good compromise between reduced execution time and stability.

**Figure 21.**
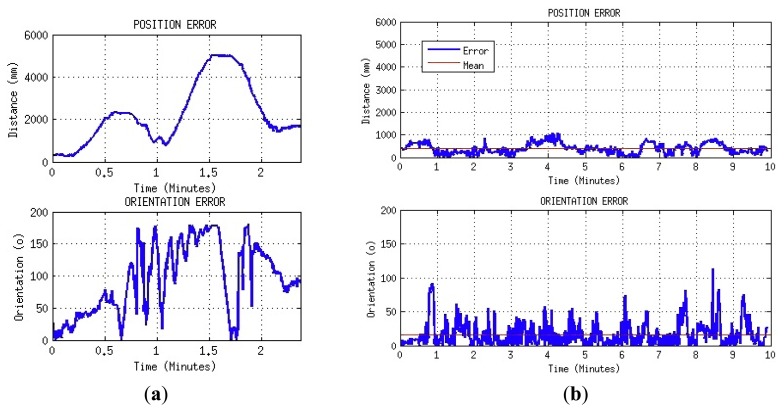
Localization tests result using: (**a**) Odometry-based system. (**b**) Modified particle filter localization system.

After this preliminary decision was taken, several tests with and without the proposed localisation procedure were performed to compare the results. The first experiment was intended to evaluate the accuracy of the position estimation using only odometry during a real game. During this test, the robot played in the same way it would in a regular match. The position and orientation errors corresponding to a real situation that occurred just two minutes after the game started are plotted in Figure 21a. As can be seen in these plots, the deviation obtained between the real and the estimated orientation grows to 180°, producing a mistaken identification of each half of the field. The estimated position also shows large errors of up to five meters prevent the robot from entering into useful play.

The same test was then executed using the proposed localization method. The results of this second test are plotted in Figure 21b. The plotted data corresponds to 10 min, the length of a half during an SPL match. In this case, the robot estimated its position with a mean error of 387 mm and orientation with a mean error of 15°.

Although the presented results may not be as accurate as those obtained by other approaches presented in Section 2, it must be taken into account that localization results do not depend exclusively on the localization method. Thus, the quality of perceptual information (such as distance estimation and the odometry system), or the number of features used for position estimation, may be improved to produce better location results. Furthermore, the provided results must also be affected by the errors produced by the arrangement of the cameras used for validation.

**Figure 22.**
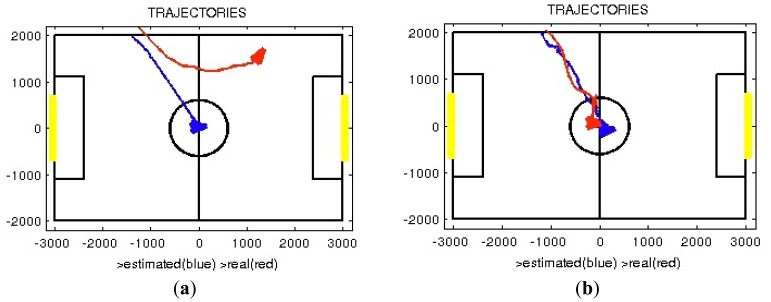
Positioning accuracy tests results using: (**a**) Path followed using odometry-based system; (**b**) Path followed using the localization system.

Another experiment was also conducted to evaluate the accuracy of the positioning system. The robot was situated on the edge of the game field and was instructed to go to the center of the game field. In the first test, the robot used its own odometry to do the walk, but in the second test, it used the modified particle filter. The results are plotted in Figure 22, respectively. In these figures the estimated trajectories followed by the robot (in blue) and the real trajectories (in red) are plotted on the game field, showing again major differences between the results of both tests.

In Figure 22a, the robot estimates a straight trajectory to the center, but in the real world the position reached is two meters and 50° from the estimation. Figure 22b shows that during the entire walk the real and estimated positions are similar (position error: 250 mm and orientation error: 17°).

**Figure 23.**
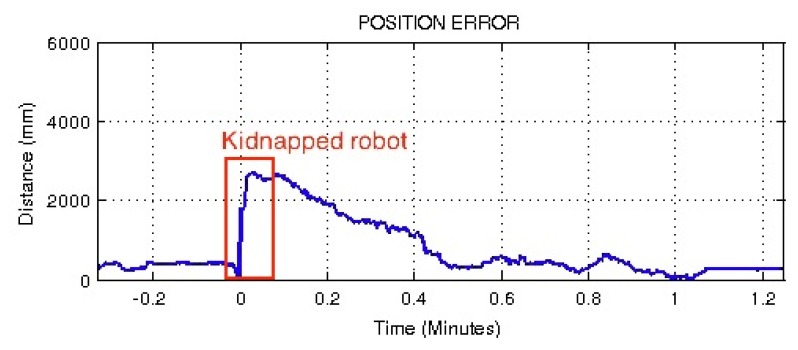
Results of the ‘kidnapped robot’ test using the proposed resampling PF (time is relative to kidnapping).

Once demonstrated the performance of the proposed localization system on the real game situation, it seems appropriate to check the performance of the filter when a ‘kidnapped robot’ situation is produced. In a new experiment, during a normal play situation, the robot was manually displaced to another position in the field that was about 3m away from the previous position. The robot used the proposed resampling PF step based on the reliability of the estimation to validate the proposed filter. Figure 23 shows that about 30 s after the kidnap, the robot had corrected its position and showed the same performance as before the kidnapping.

**Figure 24.**
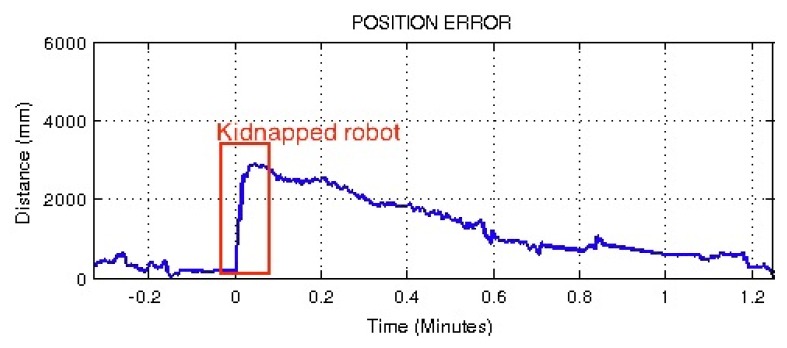
Results of the ‘kidnapped robot’ test using the classical resampling method.

The kidnapped robot situation was also repeated using a classical resampling PF step, instead of the proposed step. Figure 24 shows that the robot can recover its correct estimation, but in this case, recovery time was around one minute. The differences between these two tests can be explained by a better adaptation to the dynamic requirements of the filter shown by the proposed method in comparison with the classical approach.

## 8. Conclusions

In this article a complete procedure to implement a reliable self-localization method has been described, ranging from the perception system to the localization system itself. This localization system has been applied to Nao robots used in the SPL League, but it could be generalized for other humanoid robots.

Firstly, a sensor modeling method and the subsequent auto-adjust procedure to improve the measuring system and the perception of the environment was described. As demonstrated by the experimental results, this auto-adjust can help produce good distance estimations from features in the game field.

A new implementation of the particle filter that operated by finding the best particle in every cycle by evaluating coherence with each feature and then comparing the coherences was described. The best particle determines the sensor resetting process in case of a bad location in the game field. This filter requires a reduced number of particles in comparison with other methods, thus decreasing the CPU load.

The performance of the proposed self-localization procedure was tested. For this purpose, an auxiliary arrangement of ceiling cameras was used to obtain the real position of the robot. A study of the execution times was made to decide the optimum number of particles.

The positioning results obtained during tests show an average deviation of 387 mm in position and 15° in orientation, which gives the robot the capability of playing a whole half without becoming lost, differentiating clearly between both goals, and being aware in which side of the field it is located. The experimental positioning results also show that the robot can obtain its position with a deviation of 250 mm—precise enough to enhance strategy and avoid entering forbidden areas of the field.

The performance of the proposed filter during ‘kidnapped robot’ situations was tested—and proved to be faster than other methods. The situation was solved in only 30 s thanks to a dynamic re-sampling step that improves the estimation when reliability in the estimation decreases. Therefore, this system has proven to be an essential element for improving the game and providing a real challenge for opposing teams.

## Figures and Tables

**Table 1.** Table of execution times obtained by varying the number of particles used in the filter.

**# of particles Min.**	**Time (ms)**	**Average Time (ms)**	**Max. Time (ms)**	**Stability**
10	0. 09	0.24	1.12	Unstable
15	0.12	0.28	1.24	Unstable
20	0.14	0.29	1.35	Stable
50	0.32	0.89	2.98	Stable
100	0.67	1.71	5.94	Stable
150	0.94	2.76	7.03	Stable
200	1.29	3.73	9.29	Stable

## References

[1] Van der Molen H. (2011). Self-Localization in the RoboCup Soccer Standard Platform League with the Use of a Dynamic Tree. Bachelor Thesis.

[2] Fox D., Burdgard W., Deallert F., Thrun S. Monte Carlo Localization: Efficient Position Estimation for Mobile Robots.

[3] Thrun S., Fox D., Burgard W., Dellaert F. (2001). Robust Monte Carlo localization for mobile robots. Artif. Intell..

[4] Rubin D.B. (1988). Using the SIR algorithm to simulate posterior distributions. Bayesian Stat..

[5] Burguera A., González Y., Oliver G. (2009). Sonar sensor models and their application to mobile robot localization. Sensors.

[6] Pizarro D., Mazo M., Santiso E., Marron M., Jimenez D., Cobreces S., Losada C. (2010). Localization of mobile robots using odometry and an external vision sensor. Sensors.

[7] Payá L., Fernández L., Gil A., Reinoso O. (2010). Map building and monte carlo localization using global appearance of omnidirectional images. Sensors.

[8] Laue T., Röfer T. Particle Filter-based State Estimation in a Competitive and Uncertain Environment.

[9] Coltin B., Veloso M. Multi-Observation Sensor Resetting Localization with Ambiguous Landmarks.

[10] Thrun S., Burgard W., Fox D. (2005). Probabilistic Robotics.

[11] Barrett S., Genter K., Hester T., Khandelwal P., Quinlan M., Stone P. (2011). Sharing is Caring: Better Awareness through Information Sharing..

[12] Jochmann G., Kerner S., Tasse S., Utbann O. (2012). Efficient Multi-Hypotheses Unscented Kalman Filtering for Robust Location. RoboCup 2011: Robot Soccer World Cup XV..

[13] Bais A., Deutsch T., Novak G. Comparison of Self-Localization Methods for Soccer Robots.

[14] Tseng C.H., Chang C.W., Jwo D.J. (2011). Fuzzy adaptive interacting multiple model nonlinear filter for integrated navigation sensor fusion. Sensors.

[15] Vega J., Perdices E., Cañas J.M. (2013). Robot evolutionary localization based on attentive visual short-term memory. Sensors.

[16] Laue T., Röfer T., Gillman K., Wenk F., Graf C., Kastner T. (2011). B-Human 2011—Eliminating Game Delays..

[17] Echegoyen Z., Lopez-Guede J.M., Fernandez-Gauna B., Graña M. (2012). Visual servoing of legged robots. J. Math. Imaging Vis..

[18] Khandelwal P., Hausknecht M., Lee J., Tian A., Stone P. Vision Calibration and Processing on Humanoid Soccer Robot.

[19] Adams R., Bischof L. (1994). Seeded region growing. IEEE Trans. Pattern Anal. Mach. Intell..

[20] Laue T., Jeffry de Haas T., Burchardt A., Graf C., Röfer T., Härtl A., Rieskamp A. Efficient and Reliable Sensor Models for Humanoid Soccer Robot Self-Localization.

[21] Heikkilä J., Silven O. A Four-Step Camera Calibration Procedure with Implicit Image Correction.

[22] Tsai R.Y. (1987). A versatile camera calibration technique for high-accuracy 3D machine vision metrology using off-the-shelf TV cameras and lenses. IEEE Int. J. Robot. Autom..

[23] Zhang Z. (2000). A flexible new technique for camera calibration. IEEE Trans. Pattern Anal. Mach. Intell..

[24] Remondino F., Fraser C. (2006). Digital camera calibration methods: Considerations and comparisons. Ine. Arch. Photogramm. Remote Sens. Spat. Inf. Sci..

[25] Smith S.W. (1999). The Scientist and Engineer's Guide to Digital Signal Processing.

